# Axis-I comorbidity is linked to prospective instability of diagnoses within eating disorders

**DOI:** 10.1186/1471-244X-13-295

**Published:** 2013-11-07

**Authors:** Gabriella F Milos, Volker Baur, Sabina Muehlebach, Anja Spindler

**Affiliations:** 1 Centre for Eating Disorders, Department of Psychiatry and Psychotherapy, University Hospital Zurich, Culmannstr. 8, CH-8091 Zurich, Switzerland; 2 Division Neuropsychology, Institute of Psychology, University of Zurich, Binzmuhlestr 14/25, Zurich CH-8050, Switzerland

**Keywords:** Eating disorders, Anorexia Nervosa, Bulimia Nervosa, Comorbidity, Diagnostic instability, Affective disorder

## Abstract

**Background:**

Eating disorders (ED) are classified into Anorexia Nervosa, Bulimia Nervosa, and eating disorder not otherwise specified. Prospectively, the diagnostic instability within ED is high, but it is not clear which factors may account for this instability. So far, there is no evidence of whether psychiatric comorbidity may play a role in ED diagnostic crossover. We sought to determine possible influences of comorbidities of axis I and II on diagnostic crossover within ED.

**Methods:**

Longitudinal data of 192 female patients were collected. All patients had a diagnosis of a current ED at study entry (baseline, T0). Diagnoses were re-established both 12 months (T1) and 30 months (T2) after T0. Comorbid psychiatric diagnoses were grouped into axis I and axis II according to DSM-IV.

**Results:**

Patients with instable ED diagnoses had lifetime axis-I comorbidity more frequently than patients with stable ED diagnoses (χ^2^ = 4.74, *df* = 1, *p* < 0.05). Post-hoc exploratory tests suggested that the effect was mainly driven by affective disorders like major depression. There was no difference for axis-II comorbidity between stable and instable diagnostic profiles.

**Conclusions:**

Following previous reports of diagnostic crossover in ED, the present investigation points to an influence of a life-time psychiatric comorbidity, in particular of axis I, on follow-up diagnoses of ED. Comorbid affective disorders like major depression might facilitate a switching between clinical phenotypes. The understanding of mechanisms and causes of the symptoms fluctuation will be subject of future studies.

## Background

Eating disorders (ED) affect mainly young people, and are notably difficult to treat. While the ‘core psychopathology’ of all ED is centred on thoughts and worries about eating, shape and weight [1,2], the current ED classification is based on the following categories: Anorexia Nervosa (AN), Bulimia Nervosa (BN), and eating disorder not otherwise specified (EDNOS) (Diagnostic and Statistical Manual for Mental Disorders, 4th edition (DSM-IV) [3]. Establishment of diagnosis sets the ground for therapeutic interventions and has, thus, an important role in clinical praxis and in ED-related research. ED can have dramatic somatic, psychiatric as well as psychosocial consequences [4]. Because these illnesses have a strong tendency to last several years and become chronic [5], it is of eminent importance to meticulously observe their symptomatology and course.

Whereas AN, BN, and EDNOS share common psychopathological features, their specific characteristics arise from differential behaviour regarding food intake, compensatory behaviour, and from individual body weight [3]. These are often subject to changes during the course of the illness. A restricting eating behaviour, for instance, can switch to uncontrolled food intake followed by self-induced vomiting or use of laxatives. Further, if body weight fluctuates around the body-mass index (BMI) boundary of 17.5 kg/m^2^, the ED diagnose can repeatedly change. In fact, reports in recent years have shown that diagnostic crossover within ED over time is high [6-10]. So far, while being of importance for recognising and treating, it is not clear which are the underlying mechanisms of the diagnostic instability.

Numerous studies investigated prognostic factors influencing the course of ED (e.g., [11,12]), but rarely with regard to the diagnostic instability. In ED, the rate of axis-I (clinical syndromes) and axis-II (personality disorders) comorbidities according to DSM-IV is high [10,13-15]). In a study by Tozzi and colleagues [10], certain comorbidities of axis I and II have been elucidated to play a role in the diagnostic crossover from AN to BN and from BN to AN. A recent study by Castellini and colleagues [6] analysed the whole spectrum of ED diagnoses – AN, BN and EDNOS – with regard to axis-I comorbidity. The results of that study point to a role of mood disorders being linked to diagnostic instability. However, this work omitted to test personality disorders (axis II). Taken together, the role of psychiatric comorbidities in diagnostic crossover remains largely unclear and warrants further research.

In the present longitudinal study, we investigated diagnostic crossover in a sample of ED patients covering the full spectrum of ED diagnoses. Our aim was to examine the role of both axis-I and axis-II psychiatric comorbidity with regard to diagnostic crossover. We expected that not only the presence of axis-I comorbidities can be associated with increased probability of diagnostic crossover within ED diagnoses, but also personality disorders could play an important mediating role in the change of the ED symptomatology.

## Methods

### Sample and establishment of diagnoses

All participants received detailed information about the study, and gave written informed consent. The study was approved by the local ethics committee of the Canton Zurich, Switzerland and conforms to the Helsinki Declaration. Recruitment of participants and patient characteristics have been described in detail previously [8,16]. In brief, we initially recruited 277 female patients with ED according to DSM-IV, of whom a subset of *n* = 205 and *n* = 192 could be re-assessed in a 12-months follow-up and a 30-months follow-up, respectively. The proportions of re-assessed patients did not differ significantly between AN, BN, and EDNOS. Mean age of patients at study entry was *M*_age_ = 28.6 years (*SD*_age_ = 7.9 years), mean ED duration was *M*_duration_ = 9.3 years (*SD*_duration_ = 7.2 years). ED diagnoses at any time point (T0, T1, T2) and life-time psychiatric comorbidities were assessed using the Structured Clinical Interview for axis I and axis II (SCID I and SCID II, German version [17]) of the DSM-IV. Life-time psychiatric comorbidities were assessed at study entry (T0). These interviews were conducted by four psychologists (inter-rater reliability κ = 0.8) who never met the participants outside the interviews for the study.

### Typology of diagnostic courses

As the main aim of the present study, we assessed diagnostic stability of ED diagnoses in a 2.5-year follow-up at three time points. We classified the courses of diagnoses into three groups: stable diagnostic course, instable diagnostic course, and stable remission. A *stable diagnostic course* was characterized by the same ED diagnoses at all three time points (e.g., AN to AN to AN). Instable remission (e.g., AN to AN to remission or AN to remission to AN) was also classified as a stable pathological ED diagnostic course, i.e. no switching to another ED symptomatology. An *instable diagnostic course*, conversely, was defined as the presence of a diagnostic switch, i.e. two or more different ED diagnoses, in the course of the three time points (e.g., AN to BN to AN or BN to BN to EDNOS). Consequently, this diagnostic course also comprised cases with instable remission (e.g., BN to remission to EDNOS). *Stable remission* was present if a patient only had an ED diagnosis at the first time point (e.g., BN to remission to remission).

### Statistical analysis

To address our main study question, patients with stable remission (*n* = 25) were excluded from further analysis, leaving a final sample of *n* = 167. Patients were grouped into those with stable diagnostic course (*n* = 79, 47.3%) and those with instable diagnostic course (*n* = 88, 52.7%). For comparison of frequencies between patient subgroups (e.g., when comparing stable vs. instable with regard to axis-I or no axis-I comorbidity), chi-square (χ^2^) tests were used. Any χ^2^ expected frequency was > 5. All analyses were performed with IBM SPSS Statistics Version 20. All *p*-values were two-tailed at a threshold level of significance of α = 0.05.

## Results

### Psychiatric comorbidities: descriptive analysis (n = 192)

The large majority (*n* = 160, 83.3%) of ED patients had a lifetime psychiatric comorbidity. axis-I and axis-II comorbidities were roughly equally distributed (axis I: present in 139 patients (72.4%); axis II: present in 132 patients (68.8%). More than half of the patients had both axis-I and axis-II comorbidities (*n* = 111, 57.8%). Specific lifetime comorbid diagnoses are listed in Table 1. The distribution of ED diagnoses at any of the three study time points is listed in Table 2.

**Table 1:** Lifetime comorbid diagnoses (DSM IV)

**Axis I (clinical syndromes)**	**%**	**Axis II (personality disorders)**	**%**
**Affective disorder**	**50.5**	**Cluster A (odd)**	**9.9**
Major Depression	37.0	paranoid	5.2
Dysthymia	16.7		
		**Cluster B (dramatic)**	**17.2**
**Substance abuse**	**25.5**	Borderline	13.0
Alcohol	10.9		
Cannabis	6.3	**Cluster C (anxious)**	**55.7**
others	10.9	self-unsure	21.4
		dependent	9.4
**Anxiety disorder**	**52.1**	obsessive-compulsive	44.3
Panic disorder	10.4		
Social phobia	19.3	Not specific (appendix)	
Specific phobia	8.9	negativistic	6.3
Obsessive-compulsive disorder	31.8	depressive	21.9
Generalized anxiety disorder	14.6		

**Table 2:** Distribution of eating disorder diagnoses at any of the three studies time points

**Diagnosis**	**Study entry (T0)**	**12-month follow-up (T1)**	**30-month follow-up (T2)**
Anorexia nervosa	55	43	37
Bulimia nervosa	108	71	46
EDNOS	29	38	49
No		40	60

### Psychiatric comorbidities in patients with stable vs. instable diagnostic courses (n = 167, patients with stable remission excluded)

Patients with instable ED diagnoses had lifetime axis-I comorbidity more frequently than patients with stable ED diagnoses (80.7% vs. 65.8%, χ^2^ = 4.74, *df* = 1, *p* < 0.05). Conversely, there was no such effect for lifetime axis-II comorbidity (72.7% vs. 68.4%, χ^2^ = 0.38, *df* = 1, *p* = 0.535). Follow-up exploratory tests suggested that the axis-I effect was mainly driven by the presence of a lifetime affective disorder (Table 3).

**Table 3:** **Splitting of Axis-I lifetime comorbidities into diagnoses (follow-up χ**^**2 **^**tests, *df*** **= 1, *n*** **= 167)**

**Axis-I diagnosis**		**Stable course**	**Instable course**	**χ**^**2**^	***p***
Affective disorder	Yes	43.0%	58.0%	3.71	0.054
	No	57.0%	42.0%		
Substance abuse	Yes	20.3%	29.5%	1.91	0.167
	No	79.7%	70.5%		
Anxiety disorder	Yes	51.9%	56.8%	0.41	0.524
	No	48.1%	43.2%		

Three additional follow-up exploratory tests were also conducted: The presence vs. absence of a lifetime psychiatric comorbidity (regardless of axis) was not different between stable and instable diagnostic courses (χ^2^ = 0.89, *df* = 1, *p* = 0.345), and it made also no difference whether there were comorbidities of only one vs. both axes (*n* = 142, χ^2^ = 2.50, *df* = 1, *p* = 0.114). Lastly, within patients with axis-I comorbidities (*n* = 123), proportions of one vs. two or more lifetime diagnoses were not different between stable and instable diagnostic courses (χ^2^ = 0.67, *df* = 1, *p* = 0.415).

With regard to the influence of axis-I and axis-II comorbidity on purging/non-purging behaviour in the whole sample, we could not find any significant effects or statistical trends.

## Discussion

The results of the present study indicate a relevant role of the presence of lifetime axis-I psychiatric comorbidity regarding the stability of the ED diagnoses during an observation time of 30 months. Follow-up analysis suggested that this effect was mainly driven by the presence of lifetime affective disorders. This falls in line with two previous studies [6,10] who found an effect of the presence of mood disorder with regard to diagnostic crossover. In contrast to Castellini and colleagues [6], we did not find evidence of an effect of lifetime substance abuse on diagnostic crossover (however the number of patients with comorbid substance abuse was low in our sample).

We also expected that personality disorders may represent an important mediating role for symptoms fluctuations and, thus, diagnostic crossover within ED, but interestingly, our data do not support that view, as we did not observe an effect of axis-II comorbidities on diagnostic instability in our sample. This is in contrast to the study by Tozzi and colleagues [10] who found effects of comorbidities of both axis I and axis II on diagnostic crossover. However, effects of psychiatric comorbidities on diagnostic crossover could not be confirmed in a retrospective study by Monteleone and colleagues [7].

Because the large majority of the sample was in psychotherapy before and during the study (see [8] for details), it is important to note that some shifts might be explained by therapeutic interventions. In fact, therapy aims at changing the patient’s attitude and behaviour towards food intake, which may in the best case lead to a good outcome, but more critically also manifest as a switch between clinical phenotypes, keeping an ED diagnosis. Clinical observations show that when patients with underweight and restrictive eating behaviour are during psychotherapy under a massive pressure to gain weight, they may develop binge-eating behaviour. On the other hand, when patients with binge-eating and purging behaviour try to normalize their food intake by too rigidly restrict themselves, they may drop into underweight.

Our study points to a role of axis-I comorbidity on diagnostic crossover within ED, and herein draws attention to the presence of comorbid affective disorders, particularly major depression. What may be behind the notion that depressive symptoms may trigger ED symptom fluctuations as indicated by our data? Interestingly, a role of major depression in body weight instability and abnormal food intake has been evidenced [18]. Tozzi et al. reported that crossover between AN and BN is significantly correlated with the personality factor of self-directedness [8]. Because recent studies reported that high harm avoidance and low self-directedness predict major depression in ED patients [19], it is cogitable that low self-directedness could facilitate changes in food intake which is in turn reflected by changes in BMI. However this hypothesis has to be verified in further studies.

Anxiety disorders are very common in ED patients (e.g., [20]), but the role of anxiety disorders on the diagnostic instability is still unclear. In the present study we could not find associations between diagnostic instability within ED and anxiety disorders. Previously, we could observe that the presence of obsessive-compulsive disorders was significantly associated with a longer duration of the ED, in addition we found no difference between the prevalence of obsessive-compulsive disorder in AN compared to BN [21]. Some studies underline the important role of obsessive-compulsive traits for the course of ED (e.g., [22-24]) Anderluh and colleagues reported retrospectively that obsessive-compulsive traits in childhood were linked to a longer duration of underweight status, longer episodes of severe food restriction, and shorter duration of binge eating [22,25]. It is possible that rigidity (as obsessive-compulsive disorder or as trait) may contribute to an increased fixation of the ED symptoms and, thereby, reduced diagnostic instability.

Psychiatric comorbidities have been shown to be linked to increased symptom severity [16], and thus affect outcome [11,12,26]. Taking previous reports into account [16], the present results suggest that while the presence of both axis I and axis II comorbidities are linked to ED symptom severity, comorbidities of axis I in particular may affect diagnostic crossover within ED. The relationship between diagnostic crossover and ED outcome remains unclear. Given that diagnostic crossover is a frequent phenomenon, and ED patients often show treatment resistance, and a considerable part of patients has an ominous course of the illness, a deep understanding of the diagnostic instability could give important inputs for new treatment strategies.

### Limitations

The present study is exploratory. We applied uncorrected statistical tests, and our main result would not have remained significant after correction for multiple comparisons. Because a link between psychiatric comorbidity and symptom severity has been shown previously [16], it cannot be ruled out the possibility that symptom severity has mediated the effect between comorbidity and diagnostic instability. However, two arguments speak against an effect of symptom severity on diagnostic crossover: First, in Spindler & Milos [16], it has been shown an effect of both axis I and axis II comorbidity on symptom severity. Here, in contrast, diagnostic crossover was only significantly affected by axis-I comorbidity, and not axis-II comorbidity. We investigated the influence of axis-I and axis-II comorbidity on purging/non-purging behaviour in the *whole* sample and did not find effects. However, we did not differentiate within the AN group, although this aspect may be of interest [9].

As a final limitation, we tested our hypothesis only at the level of psychiatric comorbidities of axis I and II. We did not take into account personality factors like self-directedness or harm avoidance as was done in the studies by Tozzi and colleagues [10] and Anderluh and colleauges [25]. This has to be kept in mind, particularly as personality factors can be regarded as risk factors for the development of psychiatric disorders in general.

## Conclusions

This study draws attention to the presence of psychiatric comorbidities of axis I being linked, in a prospective view, to increased changes in the symptomatology of ED, and accordingly to diagnostic instability in ED. The study underlines the importance of a careful assessment of axis-I comorbidities in the clinical praxis, and stress the significance of a comprehensive long-term view on the ED symptomatology. Future studies may investigate how individual therapeutic strategies can be developed based on the patient’s presence or absence of psychiatric axis-I comorbidities. Furthermore, prospective studies are needed to elucidate the predictive value of symptom fluctuations for the outcome, and to define ED phenotypes.

## Abbreviations

ED: Eating disorder; AN: Anorexia nervosa; BN: Bulimia nervosa; EDNOS: Eating disorder not otherwise specified; DSM-IV: Diagnostic and statistical manual for mental disorders, 4th version; BMI: Body-mass index; SCID: Structured clinical interview according to DSM-IV.

## Competing interests

The authors declare that they have no competing interests.

## Authors’ contributions

GM and AS designed the study. AS and SM performed the statistical analyses. All authors were involved in the interpretation of the results. GM wrote the draft of the manuscript. All authors contributed to the manuscript, read and approved the final manuscript.

## Pre-publication history

The pre-publication history for this paper can be accessed here:

http://www.biomedcentral.com/1471-244X/13/295/prepub
